# Follower Ostracism and Micromanagement Leadership: The Roles of Power Threat and Gender

**DOI:** 10.3390/bs16010035

**Published:** 2025-12-23

**Authors:** Vi Phung, Cong Liu, Zhi Luo

**Affiliations:** 1 Graduate School of Applied and Professional Psychology, Rutgers University, New Brunswick, NJ 08854, USA; cong.a.liu@rutgers.edu; 2 Department of Psychology, Hofstra University, Hempstead, NY 11549, USA; zluo4@pride.hofstra.edu

**Keywords:** follower ostracism, power threat, micromanagement, gender

## Abstract

Workplace ostracism, a form of workplace harassment, delineates the experience of being excluded or ignored at work. Despite its covert nature, workplace ostracism elicits a unique pain that distinguishes it from other overt forms of harassment, such as bullying or undermining. While a growing body of literature has examined harassment directed at leaders (e.g., upward bullying), follower ostracism, in which leaders are excluded by their followers, has received relatively little attention. Drawing on Power-Dependence Theory, we conducted a multi-wave, time-lagged study (*N* = 137) to examine follower ostracism as an antecedent to destructive leadership, specifically micromanagement. The findings indicate that follower ostracism threatened leaders’ power, which subsequently motivated leaders to engage in micromanagement as a means to reestablish their influence and authority. Moreover, female leaders experience greater power threats, and exhibit more micromanaging behaviors than their male counterparts. This study advances the theoretical understanding of workplace ostracism, destructive leadership, and gender roles. It also offers practical solutions for organizations and leaders to prevent and cope with the detrimental effects of exclusion by subordinates.

## 1. Introduction

Workplace harassment, a form of workplace mistreatment, is defined as “any negative workplace interpersonal interaction that affects the terms, conditions, or employment decisions related to an individual’s job, or creates an intimidating, hostile, or offensive working environment” (Rospenda & Richman, 2004, pp. 221–222). Scholars sometimes use different labels for workplace harassment, such as workplace bullying, mobbing, abusive supervision, social undermining, and incivility (Einarsen et al., 2020; Neall & Tuckey, 2014). While harassment involves active and direct behaviors, workplace mistreatment can also manifest in passive and indirect forms, such as workplace ostracism (Ferris et al., 2017). Workplace ostracism occurs when employees are excluded or ignored at work, despite social engagement being appropriate (Robinson et al., 2013).

The definition of workplace ostracism aligns with harassment on two criteria. First, ostracism negatively affects employees’ working experience and conditions. Research has shown that ostracism is associated with reduced well-being, increased emotional exhaustion, and lower job satisfaction (Bedi, 2019). Moreover, victims of workplace ostracism often engage in interpersonal deviance (Jahanzeb et al., 2018), counterproductive work behaviors (CWBs, Zhou et al., 2021), and cyberloafing (Hu et al., 2023). Second, ostracism contributes to a hostile and threatening work environment. It reduces the sense of psychological safety at work (Noor & Abbas, 2024) and heightens the threats to four psychological needs (i.e., belonging, self-esteem, control, and meaningful existence) for ostracized individuals.

In line with these findings, the workplace ostracism literature predominantly focuses on employees as victims, examining ostracism from co-workers (Liu, 2019; Wang et al., 2022; Ye et al., 2025) and supervisors (Jahanzeb et al., 2018; Liu et al., 2023). However, much less is known about leaders as targets of ostracism, leaving a research gap in understanding how those in leadership roles perceive and respond to this subtle yet harmful form of workplace harassment.

Follower ostracism refers to scenarios in which leaders are excluded or ignored by their subordinates, positioning the leaders as victims and the employees as perpetrators. Examining follower ostracism has both theoretical and practical significance. Theoretically, it extends the existing research on workplace harassment that has primarily focused on mistreatment directed at employees (Lin et al., 2013; Liu et al., 2024; Shih et al., 2023). Practically, because leaders play a critical role in shaping employee and organizational outcomes (Li et al., 2024; Schyns & Schilling, 2013), the consequences of ostracism toward leaders may be far-reaching. When leaders feel excluded, they may exhibit maladaptive reactions that not only harm themselves but also negatively impact their employees, teams, and organizations. For example, they may engage in micromanagement, a form of destructive leadership (Erickson et al., 2015). Micromanagement, characterized as using influence in ways that harm employees or organizations (Krasikova et al., 2013), has been associated with employees’ burnout, avoidance behavior (Li et al., 2024), lower job satisfaction (Sinaga, 2025), reduced productivity (Ndidi et al., 2022), and reduced organizational commitment (Shamspour et al., 2024). Such outcomes can ultimately impede team innovation and effectiveness (Schmid et al., 2018). At the organizational level, destructive leadership has been linked to higher turnover intention, lower innovation, and decreased productivity (Mackey et al., 2021; Zhang & Bednall, 2016). Hence, it is essential to understand and prevent follower ostracism to foster healthy leadership and organizational effectiveness.

Drawing on Power-Dependence Relation Theory (PDT; Emerson, 1962), the current study examines the relationship between follower ostracism and micromanaging leadership via the leader’s power threat. Power is defined as the capacity to influence others that stems from controlling valuable resources (Lunenburg, 2012; Mead & Maner, 2012). According to PDT, power is not a unidirectional construct; instead, it is a mutual dependence between interacting parties. In organizations, leader–follower relationships are characterized by mutual dependence: employees rely on leaders for resources and guidance, while leaders depend on followers for input, performance, and support to demonstrate leadership effectiveness. When followers withdraw communication with their leaders, such as through follower ostracism, they signal reduced reliance on leaders, thus shifting the power balance. Leaders are typically motivated to maintain the power hierarchy to protect their privileged position (Maner & Mead, 2010). Follower ostracism undermines the leader’s perceived capacity to influence, and threatens their power. In the attempt to regain power, leaders may leverage their positional authority to suppress the threat by reinforcing followers’ dependence on them.

**Figure 1:**
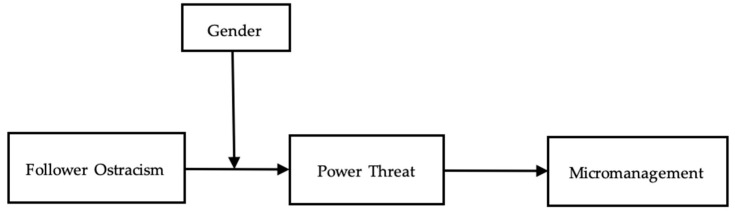
Hypothetical Research Model.

Building on this work, we predict that leaders may engage in micromanagement, characterized by excessive monitoring, directing, and prescribing how others perform their work (Allcorn, 2022). Micromanagement enables leaders to enhance their capacity for power and domination by restricting subordinates’ autonomy and increasing their dependence on the leaders. In addition, we investigate the moderating role of leader gender, postulating that female leaders, who often perceive themselves as holding less positional power (Goodwin et al., 2020), tend to experience more power threat when ostracized by their employees. The research model is presented in Figure 1.

The present study aims to make three important contributions. First, prior research has largely focused on general workplace ostracism or ostracism directed at employees by coworkers or supervisors, with little attention to situations in which subordinates ostracize their supervisors. Theoretically, this study extends the nomological network of workplace ostracism by examining ostracism from a distinct source (i.e., followers) and linking follower ostracism to leaders’ CWBs. We also contribute to workplace mistreatment research by emphasizing that, despite being perceived as more socially acceptable and less likely to be prohibited than harassment (O’Reilly et al., 2015), ostracism can nevertheless target leaders and provoke downstream destructive leadership behaviors. Practically, we offer insights for management seeking to reduce follower ostracism and prevent related harmful outcomes. Second, this study provides support to PDT (Emerson, 1962), which states that “the power of a leader over an employee is the amount of resistance of the employee which can be overcome by the leader” (Emerson, 1962, p. 32). From this perspective, follower ostracism signals low employee dependence and represents a form of resistance. Thus, we extend the power-dependence framework by highlighting power threat as the psychological mechanism linking follower ostracism to leader micromanagement. Third, this study contributes to the literature on workplace ostracism and leadership by proposing that follower ostracism has differential effects for female versus male leaders. Female leaders, who are often characterized by a relational and nurturing orientation, are perceived as less congruent with the prototypical leadership image and, subsequently, as possessing less positional power (Goodwin et al., 2020). This perspective extends PDT by showing that gender plays a critical role in shaping power dependence dynamics between leaders and followers. The present study also carries important practical contributions. As the presence of women in high-level leadership roles is steadily increasing in the U.S. workforce (Cappelli et al., 2023), understanding how gender shapes leaders’ experiences of workplace mistreatment becomes particularly relevant for management practice.

## 2. Literature Review and Hypothesis Development

### 2.1. The Relationship Between Follower Ostracism and Leader Micromanagement

Follower ostracism represents a form of upward mistreatment (Loh & Dollard, 2024; Tuckey et al., 2024). For example, employees withhold project updates from managers or deliberately ignore supervisors’ emails. By withholding social attention, employees can gain more power within the leader–employee relationship. Such behavior disrupts the balance of dependency and evokes a threat to the leader’s power (Emerson, 1962). Accordingly, leaders may be motivated to adopt behaviors aimed at reasserting authority and restoring their sense of dependency power. Research suggests that micromanagement reflects a leader’s attempt to strengthen influence and maximize power (Poornima, 2017). Micromanaging leaders intrusively monitor employees’ work and closely direct how tasks should be performed (Mishra et al., 2019). For example, they may require frequent updates, disapprove of employees’ methods, and make their performance outcomes more reliant on their own judgment.

We predict that follower ostracism increases leaders’ tendency to micromanage for three reasons. First, ostracism directly undermines leaders’ power and authority. Because leadership involves managing and directing others toward collective goals (Yukl, 1989), being ostracized undermines this influence and threatens leaders’ sense of meaningful existence within the team (Williams, 2009). In response, leaders may seek to reestablish their authority and regain visibility through micromanagement. Second, ostracism functions as a psychological stressor that elicits negative emotions such as sadness, anxiety, and anger (Howard et al., 2020). Being ostracized by employees is particularly distressing for leaders, as it disrupts communication and coordination within the team. Micromanagement, a form of destructive leadership behavior, involves excessive monitoring and demanding frequent updates (Erickson et al., 2015). By engaging in micromanagement, leaders may attempt to restore involvement and alleviate the strain of being excluded. Third, follower ostracism is a passive-aggressive form of mistreatment that can provoke counterproductive and harmful behaviors from leaders. Previous studies found that victims often reciprocate mistreatment with aggression or retaliation (Liang et al., 2022). As a result, ostracized leaders may engage in maladaptive responses to punish the follower (Wesselmann et al., 2017), using their positional authority to retaliate through micromanagement. Taken together, we predict that follower ostracism is positively related to leaders’ engagement in micromanagement.

**H1.**  *Follower ostracism is positively related to the leader’s micromanagement.*

### 2.2. The Mediating Effect of Power Threat on the Relationship Between Follower Ostracism and Leader Micromanagement

We further predicted that the psychological mechanism underlying the relationship between follower ostracism and the leader’s micromanagement is power threat. Leaders possess power over subordinates due to their legitimate authority and access to important resources and opportunities that impact employees’ career development (Litano & Major, 2016; Sturm et al., 2021). However, such power is not unilateral. Leaders also depend heavily on followers for their contributed resources, such as task updates and technical expertise, to plan, coordinate, and make informed decisions for the team (Gordon, 2011). For instance, Lundmark (2023) highlighted the importance of employees’ participation in improving decision quality and reducing decision-making time. Thus, power is inherently reciprocal and is based on the mutual dependence of involved parties (Emerson, 1962).

However, follower ostracism disrupts this mutual dependence in organizational settings. It threatens the leader’s power for two major reasons. First, exclusion signals followers’ withdrawal from reliance on the leader’s guidance and resources, hence diminishing the leader’s authority. Followers are generally perceived as more dependent on leaders due to the positional power differential (Lunenburg, 2012). Ostracizing actions, such as avoiding coordination or excluding leaders from discussion, challenge the established power hierarchy and weaken the leader’s authority. Prior evidence supports this notion: When employees have influence over how and when tasks are executed, based on their own expertise and preferences, they gain greater power in decision-making while diminishing the importance of leaders (Biron & Bamberger, 2011; Lundmark, 2023).

Second, follower ostracism impedes the flow of information that leaders rely on to effectively manage team activities. When followers withhold knowledge or updates, leaders lose access to critical insights, which impairs their ability to make informed decisions. Research shows that knowledge hiding hinders information exchange and integration, thereby impairing team performance (Chatterjee et al., 2021). Follower ostracism leaves leaders with incomplete information, thereby limiting their capacity to lead effectively. Consequently, leaders perceive diminished influence and legitimacy, which evokes a sense of power threat. Taken together, we predict that follower ostracism undermines leaders’ authority by reducing their reliance and informational access, thereby threatening their ascribed power to perform leadership duties.

**H2.**  *Follower ostracism is positively related to the leader’s power threat.*

Experiencing threats to power, in turn, will motivate leaders to engage in behaviors aimed at regaining influence. Power is crucial for leaders to allocate resources, direct activities, and influence decisions (Sturm et al., 2021). Without it, leadership cannot function effectively (Munduate & Medina, 2004). According to PDT (Emerson, 1962), leaders can reestablish power through (1) increasing control over resources to reduce dependence on employees, and (2) restricting employees’ ability to perform work independently, therefore forcing their dependence on the leader. Micromanagement serves both functions. First, by requesting frequent updates, micromanaging leaders oversee all aspects of task execution and reclaim access to information and resources that may have been withheld. Second, by dictating how tasks should be completed, leaders lower employee autonomy, task execution, and decision-making, reinforcing employees’ reliance on them.

Prior research has highlighted the connection between threats and leaders’ tendency to engage in dominant behaviors. When leaders experience threats, they develop a heightened need to affirm their authority as a means to compensate for the loss of influence. This increased need for authority is one possible antecedent of micromanagement (Tavanti, 2011). Taken together, we propose that perceived power threats are likely to drive leaders to consolidate dependent relationships in their favor through micromanagement.

**H3.**  *Power threat is positively related to the leader’s micromanagement.*

Drawing on PDT (Emerson, 1962), follower ostracism diminishes leaders’ positional power by altering established dependency hierarchies. When employees exclude a leader and start managing tasks and making decisions independently, they challenge the leader’s authority and sense of power. Power threat triggers compensatory behaviors aimed at restoring authority. Micromanagement serves this function by reinforcing employees’ dependence on the leader for guidance and approval while reducing the leader’s reliance on employees for information. Prior research has demonstrated that when leaders perceive their power to be undermined, they adopt self-serving and dominance-oriented behaviors to preserve their authority, at the expense of team goals (Maner & Mead, 2010). For example, Wu et al. (2023) found that leaders who experienced threats to their identity were more likely to exhibit abusive behaviors. Similarly, Fast and Chen (2009) demonstrated that powerholders under threat exhibited heightened aggressive and dominant responses to protect their status. Taken together, we argue that follower ostracism indirectly increases the leader’s micromanagement by evoking a threat to their power.

**H4.**  *Follower ostracism has an indirect positive effect on the leader’s micromanagement via power threat.*

### 2.3. The Moderating Effect of Leader Gender

Follower ostracism is a social stressor, and a leader’s social role shapes how they respond to it. Gender role, a specific type of social role, reflects the behavioral expectations society assigns based on gender. According to the role incongruity theory (Eagly & Karau, 2002), men are generally ascribed more dominance and authority, whereas women are associated with relational care and nurturing. These gender-based expectations influence how male and female leaders perceive and react to follower ostracism.

For female leaders, this gender role incongruity highlights the discrepancy between ideal leader images and traditional expectations of women. Female leaders’ assertive behaviors may elicit greater disapproval from subordinates. Consequently, they perceive having lower positional power than their male counterparts (Dirik, 2021). To maintain influence, female leaders rely more heavily on interpersonal trust and cooperation from their followers rather than on formal authority alone. Consistent with this aspect, Carli (1999) has noted that females possess a higher level of referent power, the degree to which others like and want to associate with them, compared to men. This form of power is particularly appealing to women because it emphasizes building and maintaining positive relationships, which aligns with traditional gender role expectations. Follower ostracism directly contradicts this strategy and undermines female leaders’ visibility and authority (Williams, 2007). Therefore, female leaders are particularly vulnerable to follower exclusion. Empirical evidence suggests that women are more attuned to disempowering behaviors that diminish their authority (Vance et al., 2004). Taken together, we predict that female leaders perceive a greater power threat when they experience follower ostracism compared to their male counterparts.

Conversely, male leaders, who are generally viewed with higher automatic legitimacy (Eagly et al., 1992), may perceive follower ostracism as less threatening. Because men hold greater legitimate power than women, they are perceived as having the formal authority to exert influence over others (Carli, 1999). Unlike female leaders, male leaders’ authority is not heavily affected by employees’ acceptance and support. Their legitimate power serves as a buffer, protecting them from the psychological costs of follower ostracism. Although male leaders may still experience informational and relational loss, their perceived authority is less dependent on employees’ recognition compared to that of female leaders.

In addition, men are generally less affected by exclusion than women. Past studies have found that prior social exclusion experiences do not impact men’s stress response (Weik et al., 2010), and they report lower levels of psychological arousal in response to social exclusion than women (Benenson et al., 2013). In contrast, male leaders’ ascribed legitimacy and lower sensitivity may buffer them from the detrimental impact of such behaviors.

Building on gendered perceptions of authority and differential responses to ostracism, we posit that gender moderates the effect of follower ostracism on leaders’ power threat. Drawing on gender differences in leadership perception and responses to exclusion, we argue that the impact of follower ostracism on leaders’ power threat is stronger for female leaders.

**H5.**  *Leader’s gender moderates the positive relationship between follower ostracism and the leader’s power threat. Particularly, the relationship is stronger among female leaders than among male leaders.*

Per H4, we predict that power threat mediates the positive relationship between follower ostracism and leader micromanagement. Per H5, we predict that gender moderates the relationship between follower ostracism and power threat. Taken together, we propose the first stage moderated mediation hypotheses:

**H6.**  *Leaders’ gender moderates the indirect positive effect of follower ostracism on micromanagement via power threat. Particularly, the indirect positive relationship is stronger among female leaders than among male leaders.*

## 3. Methods

### 3.1. Participants and Procedure

We conducted a time-lagged survey with working managers recruited via Cloud Research, an online data collection platform. Eligible participants were required to: (1) work full-time (i.e., at least 35 h per week), (2) currently hold a managerial position, (3) regularly interact with their subordinates, and (4) work on-site at least three days per week.

Following Podsakoff et al.’s (2003) recommendations on temporal separation, we administered the Time 2 (T2) survey two weeks after the Time 1 (T1) survey. A two-week interval helps minimize attrition between survey waves (i.e., participants dropping out before completing the T2 survey) while reducing common method bias associated with the same source measurements. This approach is consistent with prior empirical research (e.g., Lian et al., 2012; Mostafa et al., 2025) on destructive leadership. For instance, Lian et al. (2012) implemented a one-week time lag to examine the effects of abusive supervision on employee satisfaction and subsequent organizational deviance. Importantly, the interval aligns with our theoretical model. Williams (2009) argued that individuals immediately detect ostracism and experience threats to their psychological needs. After perceiving follower ostracism, leaders may process the information and subsequently engage in micromanagement over a short period (i.e., two weeks).

In the current research, we invited 265 participants at T1 to complete questionnaires for follower ostracism, power threat, and demographic questions. Of the 265 participants who completed the T1 survey, 164 completed the T2 survey (61.9% response rate). We excluded 27 participants who failed at least one attention check, resulting in a final sample of 137 participants. Participants were 73.70% male and 86.60% Caucasian, with a mean age of 32.25 years (*SD* = 7.86). Participants reported working at low-level (29.90%), middle-level (55.50%), and top-level (14.60%) management positions, with an average tenure of 6.25 years (*SD* = 6.61).

### 3.2. Measures

#### 3.2.1. Time 1

Follower ostracism was assessed using 10 items (α = 0.96) adopted from Ferris et al.’s (2008) Workplace Ostracism Scale. Responses were based on a 7-point Likert scale ranging from 1 (never) to 7 (always). We modified all items to specify subordinates as the source of ostracism. A sample item was “My subordinate avoided me at work.” The approach is consistent with prior studies. For example, Liu et al. (2022) measured supervisor ostracism by modifying the original items “Others avoided me at work” to “My supervisor avoided me at work.”

Power threat was measured using three items (α = 0.80) adopted from Wisse et al.’s (2019) Fear of Power Loss Scale (Wisse et al., 2019). Responses were based on a 7-point Likert scale ranging from 1 (strongly disagree) to 7 (strongly agree). A sample item was “I sometimes fear that my leadership will be undermined by my subordinates.”

#### 3.2.2. Time 2

Micromanagement was measured using 6 items (α = 0.73) adopted from Limon and Dilekçi (2021). Responses were based on a 5-point Likert scale ranging from 1 (strongly disagree) to 5 (strongly agree). We modified all items to fit with our two-week time lag by adding “In the past 2 weeks” at the beginning of each item. A sample item was “In the past two weeks, I required frequent and unnecessary status reports.”

All measures can be found within Appendix A.

#### 3.2.3. Control Variables

Prior studies have revealed that older people experience less intense reactions after ostracism than the younger group (Hawkley et al., 2011), and higher positions are associated with a higher risk for destructive leadership (Mackey et al., 2021). In this study, age was measured in years, and there were three levels of managerial positions: lower-level managers, middle-level managers, and upper-level managers.

Following Bernerth et al.’s (2018) recommendation, we conducted preliminary analyses to examine whether these variables were significantly related to the focal constructs (follower ostracism, power threat, and micromanagement). The results indicated that age, tenure, and managerial position were not significantly correlated with any focal variable (all *p*-values > 0.05). Therefore, we followed Spector and Brannick’s (2011) suggestion to exclude these variables from the final models to avoid unnecessary statistical control.

## 4. Results

### 4.1. Confirmatory Factor Analysis

We examined the discriminant validities of follower ostracism, power threat, and micromanagement (Table 1) using Mplus 8.6. A three-factor model yielded a good fit, χ^2^(129) = 213.87, *p* < 0.001, CFI = 0.94, TLI = 0.93, RMSEA = 0.05, SRMR = 0.05. The three-factor model is better than the two-factor model (power threat and micromanagement were loaded in the same factor), Δχ^2^(2) = 41.98, *p* < 0.001, and the one-factor model (all measures were loaded in the same factor), Δχ^2^(1) = 77.24, *p* < 0.001. These results provided support for the discriminant validity of the variables. Furthermore, λ (Lambda) of each item exceeded 0.60, the average variance extracted (AVE) values of three variables were all greater than 0.50, and the combined reliability (CR) values of three variables were above 0.70, indicating acceptable convergent validity (Fornell & Larcker, 1981).

### 4.2. Hypothesis Testing

Descriptive statistics, correlations, and reliability coefficients of measures are displayed in Table 2.

Following Preacher and Hayes’s (2008) bootstrapping techniques, we used the SPSS version 29.0.2, PROCESS Model 7 with 5000 bootstraps to test indirect and conditional effects. Hayes (2017) suggested that bootstrapped confidence intervals provide greater power and are robust to the non-normality of indirect effects. The findings are presented in Table 3.

Simple regression analyses revealed that follower ostracism was significantly associated with leaders’ micromanagement (*b* = 0.17, SE = 0.03, *p* < 0.001), supporting Hypothesis 1. Follower ostracism was positively related to power threat (*b* = 0.44, SE = 0.05, *p* < 0.001), supporting Hypothesis 2. Power threat was positively related to micromanagement (*b* = 0.32, SE = 0.04, *p* < 0.001), rendering support to Hypothesis 3.

Hypothesis 4 predicted a mediation effect, whereby the relationship between follower ostracism and leader micromanagement was mediated by leaders’ power threat. The results indicated a significant indirect effect between follower ostracism and leader micromanagement via leaders’ power threat (indirect effect = 0.12, SE = 0.04, 95% CI [0.03, 0.20]), supporting Hypothesis 4 (Table 4). In addition, the direct effect of follower ostracism on micromanagement was non-significant (direct effect = 0.05, SE = 0.04, 95% CI [−0.03, 0.13]), thus leaders’ power threat fully mediated the relationship between follower ostracism and leader micromanagement.

**Figure 2:**
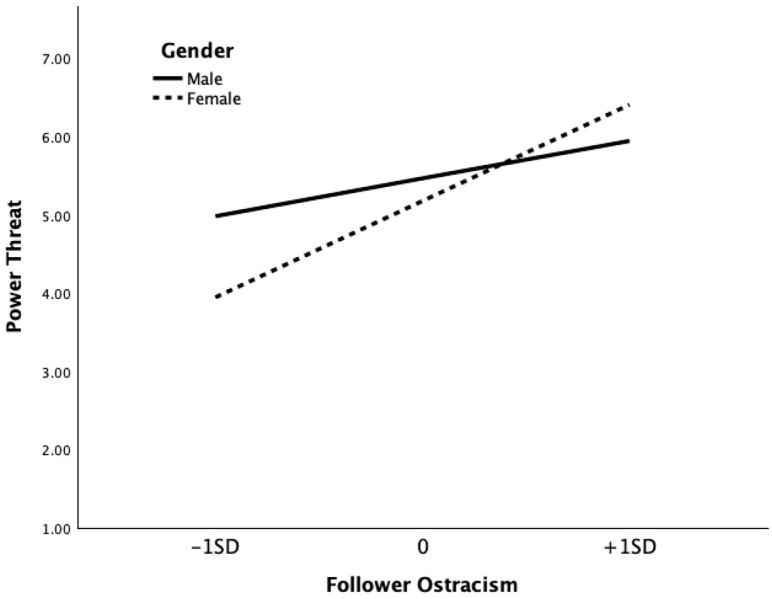
The Moderating Effect of Gender on the Relationship between Follower Ostracism and the Leader’s Power Threat.

We also found that the interaction between follower ostracism and gender was significant in predicting leaders’ power threat (*b* = 0.66, SE = 0.14, *p* < 0.001). Simple slope analysis revealed that the effect of follower ostracism on leaders’ power threat was statistically pronounced for female leaders (effect = 0.87, SE = 0.11, *p* < 0.001) compared to male leaders (effect = 0.34, SE = 0.05, *p* < 0.001). The moderating effect is presented in Figure 2. Hypothesis 5 was supported.

Hypothesis 6 proposed that gender moderates the indirect effect of follower ostracism on leader micromanagement through leaders’ power threat. The findings showed that gender moderated the indirect effect of follower ostracism on leader micromanagement, such that it was significantly stronger for female leaders (estimate = 0.24, BootSE = 0.06; 95% CI [0.09, 0.35]) than male leaders (estimate = 0.09; BootSE = 0.04; 95% CI [0.02, 0.18]). Moreover, the index of moderated mediation was also statistically significant (index = 0.15, BootSE = 0.05, 95% CI [0.05, 0.24]). Thus, Hypothesis 6 was supported (Table 4).

## 5. Discussion

This study employed a time-lagged design to examine a unique form of workplace harassment: follower ostracism of their leaders. Drawing on data from 137 leaders in the American workforce collected across two time points, we found that follower ostracism had an indirect, positive effect on the leader’s micromanagement through the leader’s power threat. Moreover, this indirect relationship was stronger among female leaders than among male leaders.

### 5.1. Theoretical Contributions

The present study makes several contributions to the literature on workplace harassment, workplace ostracism, and destructive leadership through the lens of power-dependence. First, we broaden the framework of workplace ostracism and destructive leadership by revealing an association between follower ostracism and leader micromanagement. Prior workplace ostracism research has primarily conceptualized employees as victims, focusing on supervisor ostracism (Jahanzeb et al., 2018; Liu et al., 2022) or coworker ostracism (Liu et al., 2024). This reflects the assumption that employees are dependent on their supervisors to accomplish work-related goals, and workplace ostracism increases their CWBs (Howard et al., 2020). This research contributes to prior work by highlighting that leaders also depend on their subordinates to fulfill role expectations. Follower ostracism, in turn, increases a leader’s CWBs, such as micromanagement. In line with PDT, ostracized leaders may adopt micromanagement to reinforce follower dependence and to reclaim power. This aligns with previous evidence that ostracized individuals tend to retaliate or aggress toward the source of ostracism (Ren et al., 2018; Warburton et al., 2006). Importantly, micromanagement has been shown to be highly detrimental to both employees and organizations, resulting in reduced creativity, performance, and innovation (Cleary et al., 2015; Ndidi et al., 2022). Such consequences underscore the need to understand the antecedents of micromanagement, especially in the context of follower ostracism.

Second, this study extends the literature by identifying a psychological mechanism that links follower ostracism and leader micromanagement. Specifically, we emphasize the power and dependence in the leader-follower relationship as an explanatory lens for leaders’ maladaptive behaviors. The findings align with Williams’s (2009) temporal need-threat model of social ostracism, which proposes that ostracism threatens individuals’ fundamental needs, and motivates compensatory behaviors to satisfy those needs. Follower ostracism is a psychological stressor that undermines leaders’ power, thereby eliciting destructive leadership (i.e., micromanagement) aimed at restoring power. This mechanism also aligns with recent empirical evidence suggesting that leaders who fear power loss are driven to protect it by engaging in abusive supervision (Rus et al., 2025).

Furthermore, this study advances the leadership and power framework by examining gender differences in leaders’ responses to followers. We found that female leaders experienced greater threats to power when excluded by their followers, and were more likely to engage in micromanagement than male leaders. This finding resonates with the “think manager, think male” phenomenon, which reflects the stereotype that leadership is more congruent with males (Eagly & Karau, 2002; Schein et al., 1996). The incongruity between gender and leadership explains why female leaders are more susceptible to disempowering behaviors at work (Vance et al., 2004). The results are consistent with prior research showing that women report high levels of ostracism incidents (Zimmerman et al., 2016), hence are more likely than men to interpret exclusionary behaviors as threats to their authority.

### 5.2. Practical Contributions

The current study provides several practical contributions. Follower ostracism can provoke a threat to power, and subsequent leader CWBs, such as micromanagement, if left unaddressed. To effectively manage detrimental reactions, we recommend that leaders utilize reflective tools, such as expressive journaling or mindfulness practices. Expressive writing involves documenting stressful events, focusing on one’s thoughts, emotions, and experience (Raj et al., 2025). Writing down instances of feeling left out helps leaders identify patterns in their behavioral responses, and promote more rational and deliberate actions. Prior evidence suggests that journaling improves introspective leadership, conflict resolution, decision-making, and task management (Raj et al., 2025). Mindfulness complements journaling by fostering leaders to observe their thoughts and response tendencies. Micromanagement represents a form of destructive leadership, in which leaders exercise their influence to serve their self-interest (Erickson et al., 2015). Mindfulness practice is shown to attenuate aggressive and hostile behaviors (Liang et al., 2018). It fosters leaders’ thoughtful rather than destructive behaviors in response to exclusion. These reflective techniques empower leaders to reestablish their influence through competence and respect, rather than relying on excessive monitoring and dictating subordinates’ work.

Second, we suggest that leaders facilitate regular one-on-one meetings with team members. One-on-one meetings create a space for employees to receive individualized support and build relationships that may not be achieved in other types of meetings (Flinchum et al., 2022). Maintaining consistent contact with subordinates enables leaders to reinforce their presence and influence, thereby mitigating their fear of power loss. These meetings also encourage information exchange and transparency between managers and subordinates, reducing the chance that leaders are excluded from important updates. Gaining access to individual perspectives increases leader awareness of each team member’s work processes, and reduces the tendency for leaders to respond with micromanaging behaviors.

At the organizational level, we promote integrating leadership empowerment into performance evaluation. Particularly, organizations can encourage employees to provide feedback regarding the amount of autonomy, resources, and support they receive from their leaders to make task-related decisions. Highlighting empowerment as a leadership competency encourages leaders to exercise influence through collaboration. This approach not only prevents leaders from relying on their positional power to micromanage subordinates, but also enhances shared decision-making within the team. Additionally, companies can launch recognition programs to acknowledge leaders who successfully empower their teams. Recognition (e.g., rewards, incentives, public acknowledgment) serves as positive reinforcement to validate leaders’ influence through respect, thus alleviating power threat. Empirical evidence suggests that recognition can motivate organizational citizenship behaviors, and reduce destructive responses (Yang et al., 2022). Lastly, organizations should address the root cause of follower ostracism by creating a safe environment that encourages employees to voice their concerns. Structured feedback systems, such as anonymous surveys or forums, motivate employees to share their honest thoughts without fear of retaliation or judgment. Companies can develop actionable plans based on aggregated data to ensure employees’ opinions are heard and valued. When employees perceive that their opinions are acknowledged, they are less likely to engage in defensive behaviors, such as ostracism, as a response to unmet expectations.

### 5.3. Limitations and Future Research

The current study has some limitations. First, we relied on leaders’ self-reports to measure variables of interest. Although past research has indicated that self-reports do not necessarily inflate relationships among variables, it still raises concern about common method bias (Chan, 2009). This study implements a multi-wave, time-lagged design to mitigate the risk of common method bias (Podsakoff et al., 2003). Nevertheless, future research could further strengthen causal inference by adopting multi-source designs that collect data from both supervisors and followers.

Second, our sample consisted predominantly of Caucasian and male leaders, and did not include industry information. These characteristics may limit the generalizability of the findings across organizational settings, ethnicities, and cultures. Future research should replicate the results in more diverse samples. For instance, industries that emphasize hierarchical structure, such as finance, may lead ostracized leaders to experience stronger threats to their power. Similarly, hierarchical cultures (e.g., China) strongly emphasize authority and respect for those in higher-ranking positions (Schwartz, 2006). Workplace ostracism is perceived as a violation of social norms; thus, excluded leaders develop a heightened fear of power loss. In contrast, in egalitarian cultures (e.g., Denmark), which value equality (Schwartz, 2006), leaders may interpret follower ostracism as a less direct threat to their authority.

Third, this research examined the between-person effect of leaders’ gender on the relationship between follower ostracism and leaders’ micromanagement, mediated by power threat. However, this relationship may fluctuate over time, such as on a daily or weekly basis. For example, Vahle-Hinz (2019) conducted a daily diary study to explore the spill-over effect of workplace incivility on victims’ rude behaviors on the next day. Accordingly, we highly encourage future research to implement an experience sampling method to examine both between-person and within-person effects of leaders’ responses to follower ostracism.

Fourth, follower ostracism remains a new concept that needs further attention. Although we have proposed the detrimental impact of follower ostracism on subordinates, managers, and organizations, our current study focused exclusively on micromanagement as an outcome. We highly recommend future studies to examine other individual-level, team-level, and organizational-level consequences stemming from follower ostracism. Future research could also explore how and when leaders adopt prosocial strategies to cope with follower ostracism. For instance, leaders may respond to ostracism by seeking proximity to subordinates, such as involving them in projects, scheduling informal meetings, or offering instrumental or emotional support. Leaders who place a high value on interpersonal harmony may be particularly likely to adopt such prosocial strategies rather than resorting to micromanagement (Liu et al., 2023).

Lastly, this study only examines gender as the moderator without considering other factors that influence leaders’ responses to ostracism. According to the Leader-Member Exchange (LMX) theory, leaders form in-group and out-group relationships with employees to achieve organizational goals (Graen & Uhl-Bien, 1995). In-group members are characterized by trust, respect, and a high-quality relationship, whereas out-group members exhibit more transactional relationships with lower levels of trust and support. It is plausible that the leader’s power threat and subsequent responses depend on whether the source of ostracism is ingroup or out group members. For example, being excluded by ingroup subordinates, with whom leaders trust and maintain frequent cooperation to achieve organizational goals, creates uncertainty about performance outcomes. In response, leaders may increase task monitoring of team performance. In contrast, we speculate that ostracism from the outgroup may elicit a weaker effect given the low-quality interactions between leaders and followers, unless the leaders are result-oriented. Future research can explore the moderating effect of the quality of the leader-follower relationship on the leader’s perception of threats and their subsequent behaviors when experiencing ostracism from subordinates.

## 6. Conclusions

Workplace mistreatment manifests not only in overt behaviors, such as harassment, but also in covert forms, such as ostracism. Although often overlooked, ostracism can be as harmful as, and in some cases even more damaging than, harassment. The present research demonstrates that follower ostracism threatens leaders’ sense of power, which in turn increases their tendency to engage in micromanaging leadership. Moreover, female leaders appear to be more vulnerable to follower ostracism than their male counterparts. By focusing on leaders as targets of workplace ostracism, the present study offers important insights to employees, leaders, and organizations seeking to mitigate the risks of power threat and destructive leadership resulting from follower ostracism.

## Figures and Tables

**Table 1:** Fit Statistics for Multi-Level Confirmatory Factor Analysis.

Factor Model	χ^2^/df	*p*	CFI	TLI	RMSEA	SRMR	Δχ^2^/Δdf
Three-factor model	213.87/129	<0.001	0.94	0.93	0.05	0.05	--
Two-factor model	255.85/131	<0.001	0.92	0.90	0.08	0.07	41.98 ***/2
One-factor model	333.09/132	<0.001	0.87	0.85	0.11	0.09	77.24 ***/1

Note. *** *p* < 0.001.

**Table 2:** Descriptive Statistics, Correlations, and Reliability Coefficients.

	Mean	SD	1	2	3	4	5	6	7
1. Gender	1.26	0.44	-	0.08	0.02	−0.11	0.04	−0.09	−0.00
2. Age (years)	32.15	7.86	0.08	-	0.23 **	0.02	−0.16	−0.10	−0.07
3. Tenure (years)	6.25	6.61	0.02	0.23 **	-	0.05	0.02	0.01	0.03
4. Managerial position (years)	1.85	0.65	−0.11	0.02	0.05	-	−0.10	−0.05	−0.11
5. Follower ostracism	4.86	1.42	0.04	−0.16	0.02	−0.10	(0.96)	0.61 ***	0.40 ***
6. Power threat	5.40	1.04	−0.09	−0.10	0.01	−0.05	0.61 ***	(0.80)	0.54 ***
7. Micromanagement	3.82	0.61	−0.00	−0.07	0.03	−0.11	0.40 ***	0.54 ***	(0.73)

Note. Reliability coefficients for the scales are in parentheses along the diagonal. Gender: 1 = male, 2 = female; Managerial position: 1 = lower-level managers, 2 = middle-level managers, 3 = upper-level managers. ** *p* < 0.01, *** *p* < 0.001.

**Table 3:** Results of Hierarchical Regression Analysis.

	Power Threat		Micromanagement	
	*b* (SE)	*b* (SE)	*b* (SE)	*b* (SE)
FO	0.44 *** (0.05)	0.26 *** (0.17)	0.17 *** (0.03)	
PT				0.32 *** (0.04)
Gender		0.65 *** (0.07)		
FO × Gender		0.66 *** (0.14)		
$R^{2}$	0.36	0.48	0.16	0.29
F	76.21 ***	40.39 ***	25.37 ***	56.23 ***

Note. *b* = Unstandardized regression coefficient; FO: follower ostracism, PT: power threat. *** *p* < 0.001.

**Table 4:** Conditional Indirect Effect of Follower Ostracism on the Leader’s Micromanagement.

	Power Threat	
	Effect Estimates	95%CI
Direct effect	0.05 (0.04)	[−0.03, 0.13]
Indirect effect	0.12 (0.04)	[0.03, 0.20]
Gender		
Male	0.09 (0.04)	[0.02, 0.18]
Female	0.24 (0.06)	[0.09, 0.35]
Index of moderated mediation	0.15 (0.05)	[0.05, 0.24]

## Data Availability

The data that support the findings of this study are available on request from the corresponding author. The data are not publicly available due to privacy or ethical restrictions.

## Appendix A

Items for Selected Measures

Follower Ostracism Items Indicate the extent to which the following statements applied to you at the workplace
1 = Never 7 = Always
1. My employees ignored me at work.
2. My employees left the area when I entered.
3. My greetings have gone unanswered by employees at work.
4. I involuntarily sat alone in a crowded lunchroom at work.
5. My employees avoided me at work.
6. I noticed my employees would not look at me at work.
7. My employees at work shut me out of the conversation.
8. My employees refused to talk to me at work.
9. My employees at work treated me as if I were not there.
10. My employees did not invite me or ask me if I wanted anything when they went out for a coffee break.
Power Threat Items
Indicate the extent to which the following statements applied to you at the workplace
1 = strongly disagree 7 = strongly agree
1. I sometimes fear that my leadership will be undermined by my subordinates.
2. I sometimes feel that some of my subordinates are striving for my positions.
3. I am sometimes apprehensive about my subordinates resisting my directives.
Micromanagement Items
Indicate the extent to which the following statements applied to you at the workplace in the past 2 weeks
1 = strongly disagree 5 = strongly agree
1. In the past 2 weeks, I checked on if things are done by methods dictated by me.
2. In the past 2 weeks, I monitored even the routine activities constantly.
3. In the past 2 weeks, I required frequent and unnecessary status reports.
4. In the past 2 weeks, I felt dissatisfied with acting without consulting me.
5. In the past 2 weeks, I had the worry that the subordinates will leave me in the background.
6. In the past 2 weeks, I made the subordinates feel that they were always being monitored.
